# Recurrent Ulceration and Disease at the Ileocolonic Anastomosis in Crohn’s Disease: Etiology, Prevention, and Management, a Review Article

**DOI:** 10.3390/jcm14228158

**Published:** 2025-11-18

**Authors:** Abigail Schubach, Keith Sultan, Arun Swaminath

**Affiliations:** 1 Department of Medicine, Division of Gastroenterology, Northwell Health, Manhasset, NY 11040, USA; aschubach@northwell.edu (A.S.); ksultan@northwell.edu (K.S.); 2 Department of Medicine, Division of Gastroenterology, Lenox Hill Hospital Northwell Health, New York, NY 10075, USA

**Keywords:** Crohn’s disease, recurrent ulceration, ileocolonic anastomosis, post-surgical ulceration

## Abstract

Ulceration at the neo-terminal ileum in patients with Crohn’s disease who have undergone previous resection remains a clinical challenge. While most patients will develop ulceration, it can be difficult to determine whether the ulceration represents a true Crohn’s recurrence. Scoring systems such as the Rutgeerts Score and modified Rutgeerts score can help differentiate between disease that may have higher risk for progression. Risk factors such as tobacco use, penetrating phenotype, and prior surgical resection have been identified, in addition to surgical technique. Recent evidence has supported certain surgical techniques, such as a side-to-side stapled technique and the Kono-S, to prevent post-surgical ulceration. Post-surgical prophylactic treatment with biologic therapies have the potential to reduce disease recurrence in certain patients. Post-surgical ulceration at the ileocolonic anastomosis is a complex problem that requires careful surveillance and evidence-based management. Understanding which patients are at high risk for disease recurrence and progression is important to guide treatment.

## 1. Introduction

Crohn’s disease is a sub type of inflammatory bowel disease characterized by skip lesions and transmural inflammation that can affect the entire gastrointestinal tract from the mouth to the anus. In the 1990s, an estimated 70% of patients with Crohn’s disease (CD) underwent intestinal resection for active disease [1]. With the advent of targeted biological and small molecule medical therapies, intestinal resection is required less frequently. Still, the cumulative risk of intestinal surgery in CD remains high, at about 50% to 60% after a disease duration of 10 years [2,3,4]. Unfortunately, even after surgical resection of the diseased site, recurrent ulcers and inflammation at the anastomotic site are extremely common. Among patients who’ve undergone an ileocolonic resection, recurrent lesions have been observed in the neo-terminal ileum up to 73% of the time within one year of surgery, and up to 85% within three years of surgery [5,6]. Isolated anastomosis recurrence has not always been regarded as directly Crohn’s related, though recent data, and the frequency of ulceration in Crohn’s patients suggests otherwise. Anastomotic ulcers develop much more frequently among Crohn’s patients (45–77%) compared to non-Crohn’s cohorts (1–3%) [7,8,9].

The mechanisms and risk factors for why the disease recurs after surgery are unclear. Some of the proposed mechanisms for recurrence include ischemia at the anastomotic site, decreased lymphatic flow, chronic gut dysbiosis, and type of surgical technique. Continued smoking and multiplicity of previous resections are the only risk factors that clearly have been demonstrated to influence disease recurrence [10,11,12]. Other proposed mechanisms include intestinal ischemia, surgical anastomosis types, bacterial overgrowth, lymphatic drainage disruption, absence of ileocecal valve, reaction to sutures or staples, and microscopic disease present at the site of re-anastomosis. Many trials have demonstrated that a stapled side-to-side approach leads to reduced disease recurrence when compared to a handsewn end-to-end anastomosis, making this the most widely adopted technique [13,14,15]. Newer anastomotic techniques (which treat the mesentery as a source of intestinal inflammation), such as the Kono-S, which consists of preservation of the mesentery, and contrastingly, wide mesenteric excision are also being investigated [16,17].

Differentiating between a true recurrence of Crohn’s disease and an anastomotic ulcer can be difficult. The Rutgeerts score (RS) has been developed as an endoscopic scoring system to assess the severity of recurrence of inflammation at the ileocolic anastomosis and in the neo terminal ileum [1]. The prognostic value per index score of the RS is unknown. Some studies have identified that higher indices on the RS, as well as a newly developed modified Rutgeerts score (mRS), are associated with disease recurrence [18,19]. There is an emerging body of literature which suggests that those patients with anastomotic ulcers alone are at higher risk of disease complications than those without [20].

Lastly, the pharmacologic treatment of endoscopic recurrence of Crohn’s disease remains an area of active research. Though it is generally agreed upon in consensus guidelines that patients at high risk for recurrent disease should be started on prophylactic anti-TNF therapy, there remains low quality evidence for whether this is truly effective [21,22]. Asymptomatic endoscopic recurrence is generally defined as a Rutgeerts score greater than or equal to i2, and more recently, a modified Rutgeerts score of greater than or equal to i2b; however, this has not been formally validated. While most evidence supports anti-TNF therapy for treatment of recurrent disease, there remains active research into biologics with other non-tumor necrosis factor (TNF)-based mechanisms of action, especially in patients who have prior anti-TNF exposure or failure.

## 2. Rutgeerts Score and Modified Rutgeerts Score

**Figure 1:**
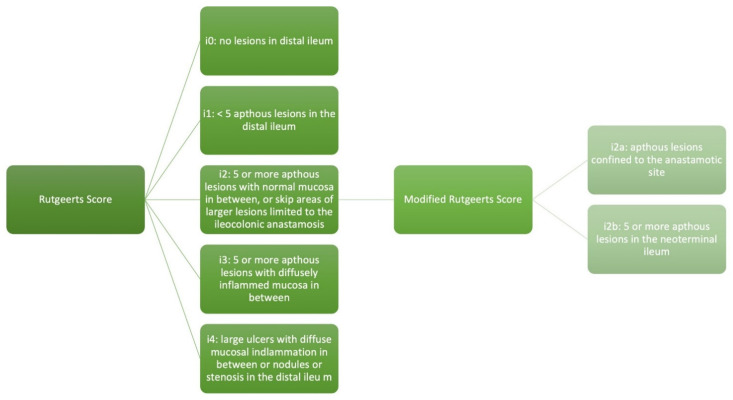
Rutgeerts score and modified rutgeerts score.

Post-operatively, recurrent lesions have been observed in the neo-terminal ileum up to 73% of the time within one year of surgery, and up to 85% within three years of surgery [5,6]. The Rutgeerts score (RS) was developed in 1990 as an endoscopic scoring system to assess the severity of recurrence of inflammation at the ileocolic anastomosis and in the neo terminal ileum [1]. The original RS stratifies the endoscopic severity into five groups (i0–i4). The score is as described: i0 (no lesions in the distal ileum), i1 (<5 aphthous lesions in the distal ileum), i2 (five or more aphthous lesions with normal mucosa in between, or skip areas of larger lesions or lesions limited to the ileocolonic anastomosis), i3 (more than five aphthous lesions with diffusely inflamed mucosa in between), or i4 (large ulcers with diffuse mucosal inflammation in between or nodules or stenosis in the distal ileum). The modified Rutgeerts score (mRS) was proposed in 2014 to differentiate i2 into lesions confined to the anastomosis (i2a) versus lesions in the neo terminal ileum (i2b) and is currently used to assess the severity of post-operative endoscopic recurrence (Figure 1) [23]. However, in a meta-analysis of seven studies published between 2008 and 2019, no difference was observed between i2a and i2b subcategories with regards to clinical or surgical post-operative recurrence [24].

High indices of the RS (≥i2) are associated with a higher risk of clinical recurrence and a re-resection when compared with a lower RS (i0–i1), though the data is somewhat conflicting [25]. Currently, patients with disease classified i0–i1 are not recommended to change treatment, while those with diffuse ileitis (i3–i4) are recommended to either begin treatment or escalate current treatment [21,26,27]. In a prospective study by Domènech et al, i2 lesions did not become symptomatic and showed a low probability to progress, 22% and 42%, 3 and 5 years after surgery, respectively [28]. Although the modified Rutgeerts attempts to further distinguish between lesions that have a higher probability to progress by distinguishing between lesions just at the anastomosis or in the neo terminal ileum, there remains conflicting evidence as to whether this distinction can truly predict disease progression. A prospective multicenter study of 225 patients found that lesions confined to the ileum (i2b) were associated with a shorter time to the development of clinical recurrence compared with lesions isolated to the anastomosis (i2a) [29]. However, two retrospective studies found no difference in clinical recurrence rates between those with i2a or i2b lesions [30,31].

While the RS and mRS are the most widely used in clinical trials as a primary outcome and in practice to guide treatment decisions, they have not been formally validated in a prospective setting, and were not designed to be used as an outcome in clinical trials. The scores also do not adjust for anastomotic configuration, because the score was based on end-to-end anastomosis, a technique which is now commonly being substituted by a stapled side-to-side technique due to better outcomes. The stapled technique consists of both inverted and everted staples, that each have drastically different impacts on healing mechanisms.

There has been major variation in endoscopic recurrence rates in CD, suggesting a discrepancy in how disease recurrence is being defined. In a meta-analysis of seventy-six studies comprising 7751 patients, the weighted means for RS and mRS were 44.0% and 41.1%, respectively, yet the variation in ER rates for RS and mRS were very wide, 5.0% to 93.0% (IQR, 29.0–59.5) and 19.8% to 62.9% (IQR, 37.3–46.5), respectively [32]. This suggests an incongruity in how the scoring system is being applied. Additionally, interrater agreement of Rutgeerts score is low, in a study of gastroenterologists who were asked to rate images of the neo terminal ileum with RS, interrater agreement was only 0.44 (95% CI, 0.24–0.62) for CD post-operative recurrence (RS ≥ i2), and 0.54 (95% CI, 0.36–0.71) for severe post-operative recurrence (RS ≥ 3) [33]. Furthermore, 13 endoscopists were asked to grade disease using the RS after watching endoscopy videotapes, and when initiation of therapy was triggered by a RS ≥ i2 12.8% (3.8–21.9) of patients were inappropriately assessed to need Crohn’s treatment [34]. The data has been conflicting; however, regarding intraobserver reliability, as a large landmark study consisting of 599 total assessments of disease activity using the RS and mRS, including 50 videos, the inter and intrarater reliability were determined to be “substantial” to “almost perfect” for both scores [35]. However, even in this study interrater reliability for aphthous ulcerations at the anastomosis or neo terminal ileum was poor, potentially reflecting a lack of consensus as to where the demarcation between anastomosis and ileum may be located.

In patients undergoing ileocecal resection with a side-to-side stapled anastomosis, both inverted and everted stapled lines are present within the same patient. The longitudinal stapled line, of both the isoperistaltic and the anisoperistaltic side-to-side anastomoses, is an inverted stapled line, while the cross-stapled line is everted. The different adaptations of the cut bowel ends determine the type of wound healing. In a study of 82 patients with CD who underwent ileocolonic resection, ulcerations were present in 63/82 [76.8%] at the inverted compared to only 1/71 [1.4%] at the everted stapled line [9]. Given that such a high percentage of patients develop ulcers at the site of their inverted staple line, this likely is interfering with grading of endoscopic recurrence. The ulcerations can be explained by a combination of secondary wound healing and ischemia induced by staple compression, and, therefore, should not be confounded with recurrence of CD. While the mRS may address a portion of this discrepancy by including lesions in the neo terminal ileum, which should not be affected by staples, some have proposed a new algorithm that ignores any ulcerations on the longitudinal staple line to avoid inappropriate management decisions.

While the RS and mRS are currently being used in guidelines and trials, more research needs to be done to standardize the scoring system used to identify which patients truly have recurrent disease and/or at risk for disease progression. The REMIND score, a newer system that consists of two separate endoscopic grading systems for anastomotic and ileal lesions, has been proposed given that patients with ileal lesions tend to have poorer long term clinical outcomes compared to patients with exclusively anastomotic lesions [29]. This data suggests that patients with ileal lesions, including mild ones (RS i1), could benefit from escalation of treatment, and, therefore, the mRS and RS may be missing a key group of patients that may be at risk. Further validation of the scoring system has the potential to both improve patient care and ensure the efficacy of treatment interventions.

## 3. Risk Factors for Disease Recurrence at the Ileocolonic Anastomosis

**Figure 2:**
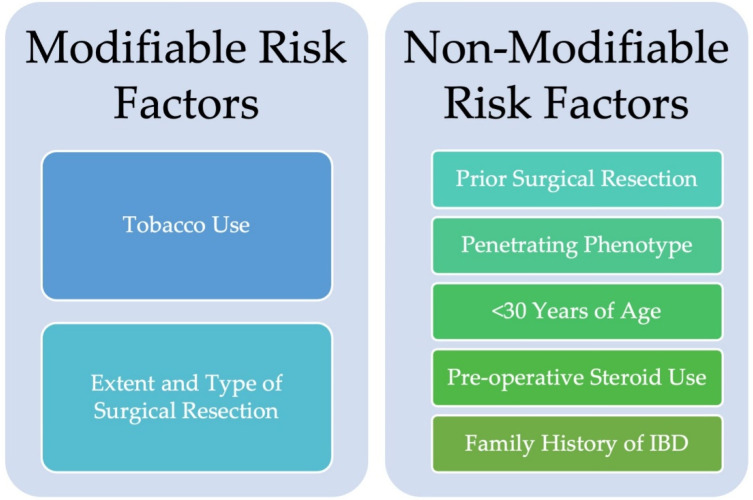
Modifiable risk factors and non-modifiable risk factors for post-operative Crohn’s disease recurrence.

Tobacco use is the most well established, modifiable risk factor for disease recurrence [10,11,12]. Many retrospective studies and meta-analyses have identified an increased rate of disease recurrence in patients who continue to smoke tobacco after surgical resection. Prior surgical resection and penetrating phenotype are also well-studied risk factors, supported by retrospective data and meta-analyses [36,37,38,39,40,41]. Other patient specific risk factors identified in retrospective studies that have conflicting data include, but are not limited to, young age at diagnosis, family history of inflammatory bowel disease, pre-operative steroid use, and both small bowel and concomitant colonic involvement (Figure 2) [12,42,43,44,45].

Tobacco, while known to have a paradoxical protective mechanism in UC, is known to contribute to the worsening of CD both pre-operatively and post-operatively. The proposed mechanisms for this association are varied. Firstly, patients who use tobacco have a reduction in mucosal cytokine levels, specifically IL-8. While the reduction in IL-8 seems to have a protective mechanism in UC patients, it is not known why this is not the same for CD patients [46]. Additionally, smoking is known to cause changes in the microvasculature, which can contribute to decreased perfusion of the colon. One mechanism to explain this phenomenon is through increased carbon monoxide concentration, which can in turn, cause chronically inflamed vasculature, resulting in ischemia and ulceration [47]. Lastly, tobacco use is known to cause dysbiosis, and specifically in patients with IBD, these bacteria produce metabolites and toxins that have the potential to dysregulate the immune response [48,49]. Therefore, it is strongly recommended to encourage tobacco cessation not only in patients with CD planned to undergo surgical resection, but in all CD patients.

One of the most well studied modifiable risk factors is method and extent of surgical resection. Potential factors related to surgical resection that may influence eventual recurrence of disease include localized ischemia and denervation, local cytokine production, and changes in the local microbiome; however, there has been a paucity of research in this area [50]. Surgical techniques vary significantly between both amount of bowel resected and type of resection and anastomosis, and more research has been dedicated to comparing eventual outcomes between the many techniques.

Surgical procedures tend to result in an increase in potentially pathogenic bacteria and a decrease in lactobacilli and bifidobacteria [51]. In patients with CD who have undergone ileocolonic resection, a reduced amount of *Faecalibacterium prausnitzii* in the ileal mucosa-associated microbiome and a greater amount of *Clostrium sensu stricto 1* have been previously associated with a higher rate of post-operative recurrence [52,53]. There has been investigation as to whether the preoperative application of probiotics change gut flora before gastrointestinal surgery, potentially improving the overall composition of microbiota [51,54]. However, these studies have not included patients with CD specifically. While the administration of probiotics changes the flora, there is not robust data to support that this change improves post-surgical outcomes.

The extent of surgical resection and mesenterectomy have also been hypothesized to affect post-operative outcomes. A trial published in 1996 demonstrated no difference between a more limited (2 cm from the margin of macroscopically diseased bowel) or extended (12 cm margin) surgical resection [55]. As the extended resection is associated with greater morbidity, generally removing the least amount of diseased small bowel that can be resected is the method performed by most surgeons. In regards to histologic resection margins, a meta-analysis demonstrated a significant increase in endoscopic disease recurrence with positive margins; however, definitions of positive margins varied significantly between the studies, making it difficult to make conclusions [56]. Additionally, it has been suggested that the mesentery may play a role in disease recurrence due to the involvement of memory T-cells in lymphatic mesenteric tissue in perpetuating the disease at anastomosis and neo terminal ileum; however, results from the SPICY trial suggests that extended mesenterectomy, when compared to mesenteric sparing in ileocolic resection, does not reduce endoscopic recurrence [57].

The most well-studied modifiable difference has been shown when comparing hand sewn end-to-end anastomosis compared with a stapled side by side technique; however, there is conflicting data. Theoretically, the side-to-side anastomosis can significantly reduce the post-operative complications due to the larger luminal diameter, and this may be the mechanism that explains why the side-to-side technique has been associated with reduced rates of post-operative complications, hospital stay, medical costs, and faster intestinal function recovery [58]. However, in patients specifically undergoing ileocecal resection with a side-to-side stapled anastomosis, a higher percentage of patients experience ulceration at the site of their inverted staple line compared to their everted staple line [9]. The ulcerations can be explained by a combination of secondary wound healing and ischemia induced by staple compression, and, therefore, should not be confounded with recurrence of CD.

While a side-to-side technique is favored in popularity, due to supporting data, there is still some conflicting evidence as to whether it truly results in better outcomes. A meta-analysis by Guo et al. analyzed 11 trials comparing anastomotic techniques again did not show a reduction in endoscopic recurrence rates with a side-to-side stapled technique [14]. However, two large meta-analysis dating to 2013 and 2014 set the stage for current standard of care in these cases. One study published by He et al., including 821 patients in eight trials, and one published by Feng et al., examining 1113 patients in 11 trials, did show a significant reduction in disease recurrence with the stapled side-to-side technique, as well as overall complications, when compared to the sewn end-to-end technique [13,59]. Because of the strong data for better outcomes, a stapled small bowel or ileocolic anastomosis is now recommended over a sewn end-to-end in some guidelines; however, some guidelines rely on surgeon preference because of the limited amount of data available to support either approach [15,60].

A newer resection technique is now being studied called the Kono-S anastomosis. This technique, first reported in 2003, is thought to reduce disease recurrence because the stapled ends of the bowel are left in place as a “supporting column” serves as a supportive backbone of the anastomosis to help prevent distortion of the anastomotic lumen, and the innervation and blood supply is preserved due to the anti-mesenteric orientation. Lastly, the isoperistaltic orientation allows for post-operative endoscopy. In a randomized clinical trial, the SuPREMe-CD study, published in 2020, 79 patients were randomized to Kono-S or stapled side-to-side ileocolic anastomosis, and after 6 months, the endoscopic CD recurrence rates were 22.2% vs. 62.8% in the Kono-S and control groups, respectively (*p* < 0.001) [16,61]. This trial is promising; however, conflicts with prior retrospective data suggesting that wide mesenteric excision was associated with reduced rates of disease recurrence and reoperation rates [17,61]. More clinical trials are currently underway to assess the benefit of Kono-S, which are needed to confirm the benefit of this technique before widespread adoption. Promisingly, there are two trials underway to compare all of the techniques mentioned above, entitled the End2End in the Netherlands and HAND2END in Italy [62].

## 4. Proposed Mechanisms for Disease Recurrence

In non-Crohn’s cohorts, the anastomotic ulcers are rare and typically attributed to mechanical or ischemic factors, whereas in Crohn’s cohorts, these ulcers may be associated with disease recurrence or post-operative healing phenomena. In a ferret model, induced ischemia was achieved in isolated loops of small intestine and resected; 2 weeks later, chronic transmural inflammation, ulceration, and granuloma formation were identified at the anastomotic site in the bowel; however, no changes were seen in the control group. This suggests that perhaps ischemia can induce Crohn’s disease [63]. Though, some data suggests, that what has been endoscopically determined to be ischemia, is truly just recurrent Crohn’s disease. A retrospective study supports this, where subjects were selected at random from a retrospective database of patients with Crohn’s disease and who had undergone an ileocolic resection with subsequent endoscopic assessment of the anastomosis and neo-terminal ileum. Twenty-seven of the twenty-nine histology specimens had evidence of CD-like features. In contrast, only two specimens, accounting for two of eight patients, had histologic features of ischemia, and both specimens also had Crohn’s-like features [64].

Decreased lymphatic flow has also been proposed to influence disease recurrence, as decreased lymphatic density in the mucosa and submucosa has been associated with a high risk of endoscopic recurrence, and a high density a predictor of non-recurrence [36]. However, low lymphatic density has also been proposed as a consequence of disease rather than predictor post-operatively [65]. Additionally, mesenteric nerves are implicated in the pathogenesis of CD. Plexitis, defined as the presence of at least one inflammatory cell in an enteric ganglion or nerve bundle, in the resected ileocolonic specimen, is also a predictive factor for early endoscopic recurrence [66,67]. Interestingly, plexitis is more commonly found proximal to the anastomosis, which is where recurrent disease is most often found, suggesting this may play a role in the pathophysiology.

Lastly, gut dysbiosis, consisting of certain enriched bacteria, have been associated with a higher risk of disease recurrence [68,69]. IBD patients’ gut microbiomes tend to be enriched with certain bacteria at a higher rate, and have a reduction in bacterial diversity, compared to healthy controls. After ileal resection, endoscopic recurrence is associated with bacteria that are reminiscent of those observed generally in ileal CD compared with healthy subjects. Specifically, an increase in Proteobacteria phylum and a decrease in several members of the Lachnospiraceae and the Ruminococcaceae families within the Firmicutes phylum have been used to predict relapse at a more accurate rate than clinical factors [68]. Additionally, in an analysis of a large prospective, well-characterized cohort of 130 Crohn’s disease patients longitudinally from the time of surgery (POCER study), dominance of Enterobacteriaceae at baseline and 6 months post-operatively was associated with an increased risk of disease recurrence at 18 months, while dominance of Lachnospiraceae was associated with diminished inflammation via anti-inflammatory short-chain fatty acid production [69]. The luminal microenvironment is likely to play a key role in either the preservation or degradation of healthy mucosa post-surgery.

## 5. Surveillance

Current guidelines recommend a post-operative surveillance program incorporating endoscopic surveillance to detect recurrence after ileocolonic resection [21]. This is because most recurrent disease is clinically silent [5]. Even 8 weeks post-resection, studies have shown microscopic recurrence of inflammation [70]. Recurrent lesions have been observed in the neo terminal ileum up to 73% of the time within one year of surgery, and up to 85% within three years of surgery [5,6]. All patients are recommended to undergo ileocolonoscopy at 6 to 12 months after resection. There are no formal guidelines and not enough evidence to recommend how often to survey patients after the initial post-operative endoscopy; however, in patients who choose a surveillance approach over starting prophylactic pharmacologic therapy, routine surveillance in timed intervals is important to closely monitor for recurrence. It may be prudent to also perform routine endoscopy in patients with higher risk for recurrence, including younger patients, those with prior surgeries, and with penetrating disease, though there is not necessarily evidence to support this practice. With more prompt discovery of recurrent disease, the earlier medical treatment can be initiated to prevent disease progression and a second surgery.

## 6. Post-Operative Prevention of Recurrent Disease

Evidence as to whether to start primary prophylaxis for Crohn’s disease after a surgical resection is conflicting. Generally, in high-risk patients, anti-TNF therapy is recommended, given the potential benefits likely outweigh the risks. The American Gastrointestinal Association (AGA) suggests pharmacologic prophylaxis with anti-TNF therapy and/or thiopurine therapy in high-risk patients post-operatively or among those who have recurrence of i2 or greater at their 6–12 months post-operative colonoscopy. The American College of Gastroenterology (ACG) also recommends prophylaxis with anti-TNF in combination with thiopurine therapy in high-risk patients, albeit both recommendations come with low quality evidence [21,22]. High risk patients are characterized as patients who are less than 30 years old, have active tobacco use, greater than or equal to two prior surgeries for penetrating disease with or without perianal disease, while low risk patients are generally older than 50 years of age, non-smokers, and have a disease duration of over 10 years. The AGA and ACG specifically recommend against the use of mesalamine due to poor evidence to support its use.

The trial data for anti-TNF therapy post-operatively has been somewhat mixed, with more data supporting the prevention of histological disease recurrence over endoscopic and/or clinical recurrence. Yet, it remains the pharmacologic therapy with the most data to support its use post-operatively. In a trial of 51 patients randomly assigned to receive adalimumab, azathioprine, or mesalamine after ileocolonic resection, both endoscopic and clinical recurrence were significantly reduced in the adalimumab group compared to the others [71]. In another randomized trial of 24 patients, half were prescribed infliximab after ileocolonic resection, there was a non-significant higher proportion of patients in clinical remission in the infliximab group, yet the histologic recurrence rate at 1 year was significantly lower in the infliximab group [72]. In another randomized prospective trial, 31 who had ileocolic resection were randomly assigned to infliximab or no treatment. The infliximab group achieved higher endoscopic remission at 12 months, 78.6% vs. 18.8% (*p* = 0.004). Although the clinical remission scores between the two arms were not significantly different at 12 or at 36 months [73], there is an often a longer lag between endoscopic appearance of inflammation and the onset of clinical symptoms. Finally, in one of the largest landmark trials of 297 patients randomized to infliximab or placebo, infliximab was superior in reducing endoscopic recurrence, based on Rutgeerts scores ≥ i2 [74].

Anti-TNF therapy has been shown to be largely superior to thiopurines in as primary prevention of disease recurrence in meta-analyses, though direct comparison of the two therapies are limited to small trials, and these have shown no difference. Generally anti-TNF therapy is recommended over thiopurine therapy due to the more robust data supporting the prevention of recurrent disease for anti-TNF therapy. In a randomized 52 week trial of 91 patients comparing the effects of adalimumab + metronidazole with azathioprine 2.5 mg/kg + metronidazole on endoscopic recurrence at one year, no statistically significant differences between the groups were found [75]. However, in a randomized trial of 51 patients comparing adalimumab to azathioprine to mesalamine, both clinical and endoscopic recurrence rates were significantly reduced in the adalimumab group. In a landmark study of 279 patients randomized to placebo or infliximab therapy 5 m/kg over a period of 76 weeks, there was no statistical difference in the primary endpoint (composite of CDAI increase > 70 pts and endoscopic recurrence > i2), though endoscopic recurrence was significantly reduced in the anti-TNF group [74]. In a meta-analysis including six pooled randomized controlled trials comparing thiopurines and anti-TNF agents, a superior effect was demonstrated for anti-TNF agents compared with thiopurine for the prevention of endoscopic post-operative recurrence (POR) defined as Rutgeerts’ score ≥ i2 (relative risk, 0.52; 95% CI, 0.33–0.80) and also clinical POR (relative risk, 0.50; 95% CI, 0.26–0.96) [74,75,76,77,78,79,80].

While the data from the small trials are somewhat conflicting, when looking at the pooled data anti-TNFs appear to be superior when compared to thiopurines at preventing both endoscopic and clinical POR. Evidence for newer biologic agents are somewhat limited due to their more recent introduction, yet there remains a high number of gastroenterologists prescribing these as primary prophylaxis [81]. Retrospective data for vedolizumab has been mixed, with studies showing both comparable post-operative endoscopic recurrence rates to anti-TNF therapies, but also an increased risk of endoscopic recurrence [65,66]. In the recently published first randomized placebo-controlled study of vedolizumab post-operatively compared to placebo, endoscopic recurrence (Rutgeerts’ score ≥ i2b) was observed in 10 of 43 patients in the vedolizumab group versus 23 of 37 patients in the placebo group (difference −38.9% [95% CI −56.0 to −17.3]; *p* = 0.0004) [82]. This suggests that vedolizumab may be a reasonable choice; however, there is more robust trial data to support anti-TNF use.

There remains no randomized trial data for ustekinumab in the preventative setting, though retrospective data suggests similar efficacy to vedolizumab in prevention of endoscopic recurrence [83]. None of these studies specifically addressed only patients with disease limited to the anastomosis (i2a).

Overall, most of the data currently supports using anti-TNF therapy with or without a thiopurine for CD patients post-operatively in high-risk patients. While the use of newer biologic therapies is becoming more common, there currently is no robust data to support their use yet, mostly due to their more recent development. More research must be done to compare therapies post-operatively to help guide clinicians as to how to best prevent post-operative disease recurrence.

## 7. Conclusions

Recurrent Crohn’s disease after ileocecal resection remains a difficult management challenge. Post-operative recurrence specifically in the neo terminal ileum, and its various risk factors, has been more closely studied. Modifiable risk factors, such as tobacco use, as well as non-modifiable risk factors, such as penetrating phenotype and prior resection, are known to increase this risk. More research is currently being conducted to identify specific biomarkers that may help predict which patients are at highest risk of recurrence [84]. While the modified Rutgeerts’ score can help standardize ulceration extent and severity, more data to guide its use and validation must be conducted to ensure it is being used to make informed treatment decisions. Isolated anastomotic ulcers are more common in IBD patients than in other populations that require this same surgery and appear refractory to medical therapy. There is, to date, no specific endoscopic therapy or medical therapy that has proven to prevent or reverse isolated anastomotic ulcers. Evolving surgical techniques, such as the Kono-S, have the potential to improve outcomes. Lastly, while anti-TNF therapy has data to support its use as primary prophylaxis against recurrent disease, newer biologic therapies need more randomized trial data to support their use in this setting.

## Abbreviations

The following abbreviations are used in this manuscript:

CD	Crohn’s Disease
UC	Ulcerative Colitis
IBD	Inflammatory Bowel Disease
RS	Rutgeerts Score
mRS	Modified Rutgeerts Score
anti-TNF	Anti-tumor necrosis factor
